# Development and Implementation of a Radiation Safety Screening Protocol for a Clinical Study Using Amyloid Positron Emission Tomography Imaging

**DOI:** 10.31486/toj.25.0095

**Published:** 2026

**Authors:** Tamara G. Fong, Madeleine Martine, Cole Heine, Yonah Joffe, Julianna Liu, Mackenzie Topper, Eva M. Schmitt, Phillip H. Kuo, Carrie A. Redlich, Sharon K. Inouye

**Affiliations:** ^1^ Aging Brain Center, Hinda and Arthur Marcus Institute for Aging Research, Hebrew SeniorLife, Boston, MA; ^2^ Department of Neurology, Beth Israel Deaconess Medical Center and Harvard Medical School, Boston, MA; ^3^ TTi Health Research & Economics, New York, NY; ^4^ Zucker School of Medicine, Hofstra University/Norwell Health, Hempstead, NY; ^5^ Department of Clinical and Health Psychology, University of Florida, Gainesville, FL; ^6^ University of Rochester School of Medicine and Dentistry, Rochester, NY; ^7^ Department of Radiology, City of Hope National Medical Center, Duarte, CA; ^8^ Yale Occupational and Environmental Medicine Program, Yale School of Medicine, New Haven, CT; ^9^ Department of Medicine, Division of Gerontology, Beth Israel Deaconess Medical Center and Harvard Medical School, Boston, MA

**Keywords:** *Patient safety*, *positron-emission tomography*, *radiation exposure*, *radiation monitoring*

## Abstract

**Background:**

Amyloid positron emission tomography (PET) is gaining popularity for clinical and research purposes, particularly since the US Food and Drug Administration approval of anti-amyloid therapies. While the benefits of PET imaging in clinical care outweigh the risks associated with radiation exposure, the risks from elective radiation exposure in research should be carefully considered. Currently, no published or widely used guidelines consider prior radiation exposure as part of the eligibility determination for prospective participants in a clinical trial that includes ionizing radiation exposure.

**Methods:**

We reviewed the medical literature and current studies listed on ClinicalTrials.gov for radiation safety criteria and study protocols that include amyloid PET scans. We then developed a safety screening procedure to systematically estimate prior radiation exposure to determine eligibility for participation in an amyloid PET substudy and implemented this procedure in the Successful AGing after Elective Surgery (SAGES) study.

**Results:**

Of the studies including amyloid PET that were listed on ClinicalTrials.gov (n=92), prespecified exclusion criteria with specific amounts of radiation exposure were provided in only 1% of studies; 37% of studies did not report any screening for prior radiation exposure. Using the screening protocol we developed for the SAGES study, 17% of 101 participants were deemed ineligible for the amyloid PET procedure because of prior exposure.

**Conclusion:**

A systematic, standardized screening protocol to determine the prior radiation exposure of potential participants should be used as a tool in clinical studies involving elective radiologic procedures to minimize risk to participants.

## INTRODUCTION

Imaging that involves exposure to ionizing radiation is being used increasingly in clinical trials to determine study eligibility, monitor treatment response, and contribute to our understanding of disease pathophysiology.^1,2^ These types of procedures are routinely used in the practice of clinical medicine, and the medical necessity and benefits of diagnosing or treating health problems often outweigh the risks associated with the exposure. When imaging is used solely for research purposes, however, the risks of radiation exposure should be carefully considered, especially for research studies that may not directly benefit the participant. In human subjects research, the investigator has the responsibility to adhere to the principle of beneficence and ensure that participants are not harmed. Risks must also be adequately disclosed to participants, and the benefits to the individual and/or society must ultimately outweigh the risks. While the International Atomic Energy Agency (IAEA) does not impose dosage limits on healthy patients, the IAEA Biomedical Research Involving Radiation Exposure website states “…appropriate dose constraints should be authorized for each research programme and the protection should be carefully optimized. For research projects involving the use of ionizing radiation, the ICRP [International Commission on Radiological Protection] and WHO [World Health Organization] recommend the use of categories of risk arranged according to the radiation dose and estimated level of risk to be received by the subject.”^3^ Exposure to ionizing radiation in a research study falls under the purview of the institutional review board overseeing that particular study. However, widely used guidelines that consider an individual's history of exposure to ionizing radiation when determining eligibility for participation in clinical studies are lacking.

On average, Americans are exposed to about 0.62 rem (roentgen equivalent man), or 6.20 mSv, of radiation each year.^4^ The sievert (Sv) and the millisievert (mSv), measures of ionizing radiation that account for the type of radiation and the sensitivity of tissues and organs, can be used to estimate the potential for causing harm. Approximately half of radiation exposure comes from natural background radiation, while the other half is accumulated through man-made sources, most commonly medical imaging.^4^ Mammography and plain radiographs (eg, dental or chest x-rays) expose patients to relatively small amounts of radiation per procedure (0.1 to 0.4 mSv),^5,6^ whereas computed tomography (CT) and nuclear medicine imaging expose patients to much higher amounts of radiation (<1 to 41 mSv).^6^ For hybrid imaging modalities such as amyloid positron emission tomography (PET) scans, the radiation is estimated to be in the range of 4 to 7 mSv from the injected radiopharmaceutical,^7^ and the accompanying CT scan used for localization further increases the amount of radiation exposure by an additional 2 to 4 mSv.^8^ High mSv radiation exposure also occurs during tests such as a cardiac perfusion stress test (2 to 41 mSv)^6,9-11^ or cardiac angiography (4 to 32 mSv).^6,9-11^

Previously, the estimation of cancer risk associated with radiation was extrapolated from studies of atomic bomb survivors exposed to single, high-level radiation exposure (>100 mSv) across the whole body during a very short period, but whether these estimates apply to chronic, low-dose exposure is unclear.^12^ The linear no-threshold model^13^ has been proposed as a way to estimate the risk of radiation-induced cancer based on the following assumptions: all exposure to ionizing radiation is harmful; there is a linear dose-response, even for very low doses, where biologic effects are difficult to study because of statistical limitations; and the effect of radiation exposure is cumulative over the lifetime.^14^ Epidemiologic studies support this model and have shown incremental increases in cancer risk even with low-dose radiation exposure (<100 mSv).^15-17^ For example, a 2025 study based on current utilization and radiation dose levels estimated that CT examinations might account for 5% of all new cancer diagnoses per year.^18^ This finding is of particular concern, especially given that imaging studies are increasingly being used clinically^19^ and in research.^20-22^ Studies have also found that relatively high numbers of patients have a high cumulative radiation exposure.^23,24^ Currently, the general practice around radiation safety is to follow the “as low as reasonably achievable” principle to minimize risks. The current US Department of Energy limit for occupational exposure to radiation is a total effective (whole body) dose of 50 mSv per year,^25^ and the US Food and Drug Administration (FDA) Radioactive Drug Research Committee recommends exposure limits from a single study or cumulatively across numerous studies (total dose) to be <50 mSv per year for an adult research subject.^26^

Advanced imaging modalities have substantially aided the study of brain function and pathology, with a growing number of PET ligands now being used to investigate various conditions in clinical and in research settings.^2^ For example, florbetapir F-18 PET imaging is used to quantify and localize beta-amyloid pathology with high diagnostic accuracy for Alzheimer disease.^27-30^ With the development of Alzheimer disease drugs that rely on precise diagnosis and quantification of beta-amyloid pathology,^31,32^ the use of amyloid PET scans is rapidly growing. However, cumulative doses that patients receive across all imaging modalities for clinical care and during research participation are not routinely quantified. Tracking exposure amounts from medical imaging is challenging, as different machines deliver different amounts of radiation, and the dose absorbed depends on size, weight, and the part of the body targeted by the radiation. Among clinicians, awareness of the importance of considering cumulative radiation exposure when ordering CT examinations is increasing, as is support for regulatory mechanisms to manage radiation risk and enhance patient safety.^19,33^

Considering this background, the aims of this study were to focus on the single imaging modality of amyloid PET scan and to (1) review the literature and the studies listed in ClinicalTrials.gov and describe current procedures used to screen potential participants for prior radiation exposure in clinical trials involving amyloid PET scans; (2) detail the screening tool we developed to assess cumulative radiation exposure from radiologic procedures for use in our prospective cohort study of postoperative delirium, the Successful AGing after Elective Surgery (SAGES) study; and (3) demonstrate the application of this tool in an amyloid PET study we conducted. Ultimately, we hope that investigators conducting clinical trials with ionizing radiation exposure who wish to include pre-enrollment safety screening might use our approach as a starting point that can be adapted to fit their specific needs.

## METHODS

### Reviewing the Literature and ClinicalTrials.gov

To find radiation safety guidelines and procedures, we conducted a review of the literature in PubMed, followed by a review of the ClinicalTrials.gov database (Appendix 1).

### Developing a Radiation Safety Screening Tool

We utilized expert clinical judgment of the study investigators, evidence from our review of the medical literature, and safety criteria described in ClinicalTrials.gov to develop a systematic screening tool for assessing prior radiation exposure. First, we divided clinical radiologic procedures into 4 categories by modality: (1) standard radiographs and diagnostic radiology (ie, fluoroscopy and dual-energy x-ray absorptiometry [DEXA] scan); (2) CT; (3) interventional radiology; and (4) nuclear medicine procedures. We listed common radiologic procedures, such as chest radiographs and head CT scans, under the applicable category to create a structure that would prompt the participant about prior exposure.

We consulted published data listing amounts of radiation exposure from common medical procedures^5^ to create a reference table of radiation levels measured in mSv for each procedure. Radiation values were widely variable, ranging from 0.005 mSv for dental radiographs to 70 mSv for a transjugular intrahepatic portosystemic shunt placement. While US Department of Energy and FDA guidelines set a 1-year radiation limit of 50 mSv, we opted to set our screening threshold at 30 mSv to minimize the risk of participation in a voluntary research study involving radiation and to account for potential imprecision in self-reporting. We developed a form in REDCap^34,35^ for inputting the radiologic procedures from the prior 12 months reported by the study participant, and the amount of radiation exposure was calculated automatically (Appendix 2).

### Screening for Radiation Exposure in the SAGES Study

The SAGES study is a longitudinal cohort study of adults ≥70 years (n=560, SAGES I) designed to examine novel risk factors and long-term outcomes associated with postoperative delirium.^36^ During an extension of the project, a second cohort (n=420, SAGES II) was recruited to examine the relationship between delirium and Alzheimer disease and related dementias.^37^ One of the principal hypotheses examined in the SAGES study was whether underlying Alzheimer disease pathology increased brain vulnerability to stressors associated with hospitalization, surgery, and anesthesia, subsequently leading to delirium and long-term cognitive decline. For this reason, amyloid PET imaging was done in a subpopulation of the SAGES cohort to determine the presence of underlying Alzheimer disease pathology. Two study investigators leading the amyloid PET substudy were the principal developers of the radiation safety screening tool. After the screening tool was developed, 6 other lead SAGES study investigators reviewed the tool for content validity.

The radiation screening flow chart is presented in the Figure. First, a trained research associate asks the participant to recall any radiation treatment for cancer received in the past 5 years. The study physician reviewed participants who reported a history of cancer radiation treatment within the past 5 years, and if exposure was confirmed, the participant was deemed ineligible for the study. If the patient reported no history of cancer or had been treated with radiation more than 5 years ago (confirmed by the study physician), screening continued. Participants were asked to recall any radiologic procedures received in the past 12 months. The research associate entered the types of procedures, number of procedures received, and procedure dates into the screening tool (Appendix 2). To help ensure complete information was obtained, the research associate asked participants specific questions about the major categories of radiologic procedures (eg, “Have you had any x-rays in the past year?”). If a participant answered “yes” or “don’t know” to any of the major category questions, they were asked to respond “yes” or “no” to a standardized list of procedures. The research associate entered each radiologic procedure and the number of times the procedure was completed, and the tool calculated a “Total Dose (mSv)” for each participant. The screening process took a maximum of 15 minutes to complete.

**Figure.**
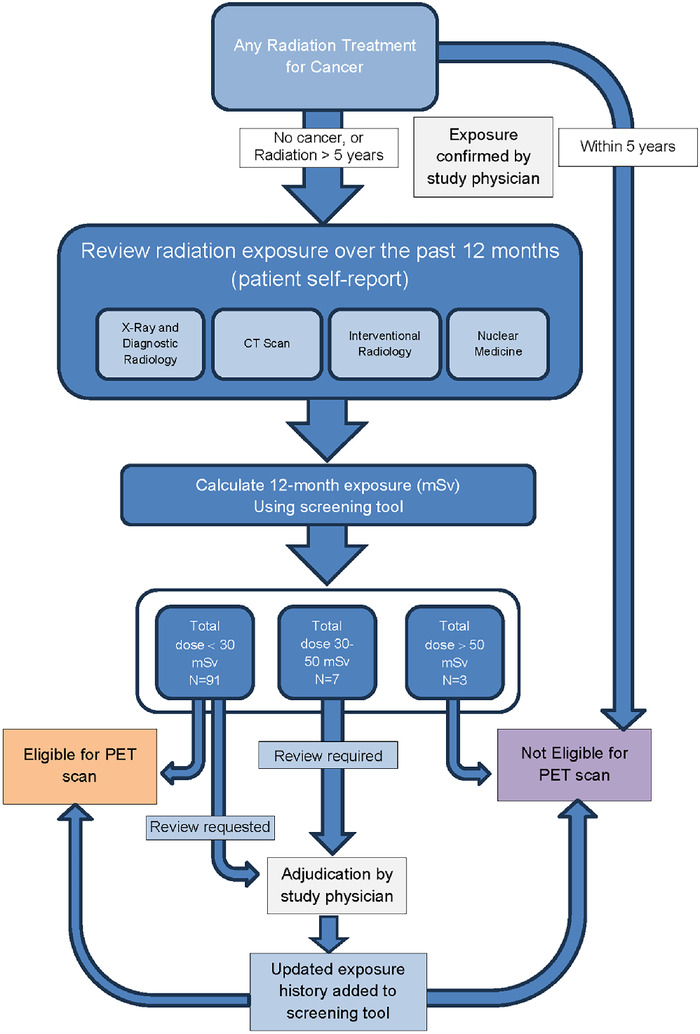
**Radiation screening flow chart.** First, participants are asked if they have received radiation treatment for cancer (top box, pale blue). If the answer is “yes,” the study physician reviews the participant's records. If radiation treatment is confirmed to have occurred within the past 5 years, the participant is excluded from participation (purple box). Participants who have not had radiation treatment for cancer or who received the treatment more than 5 years ago are asked if they have undergone x-ray and diagnostic radiology, computed tomography (CT) scans, interventional radiology, and/or nuclear medicine procedures in the prior year (second rectangle, darker blue). If the answer is “yes” to having undergone any of these procedures, the participant is asked to identify the specific procedures and how many times the procedure was done. For participants who reply “don’t know,” a list of possible procedures is read to the participant, who then responds with “yes” or “no” to each procedure. Any procedure with a “yes” response is entered on the screening tool (Appendix 2). A total dose is then calculated and used to determine eligibility for positron emission tomography (PET) scan. For participants who require further radiation exposure review because their total dose is 30 to 50 mSv (millisievert) or because the research associate requested review (ie, the patient did not recall specific procedures received), radiologic reports from the medical records are requested and adjudicated by the study physician (grey box). Procedures listed in the medical records and procedures not previously recorded are entered on the screening tool, and a new total dose is calculated. For any uncertain cases, the study physician may review the case with a second study physician for adjudication.

Participants with a 1-year cumulative radiation exposure <30 mSv were eligible to undergo an amyloid PET scan without further review, and those with a 1-year cumulative total dose >50 mSv were ineligible. Participants with a 1-year exposure between 30 and 50 mSv underwent further review by one of the study clinicians. Because radiation treatment for cancer (definitive or adjuvant) may involve moderate to high levels of radiation exposure, we reviewed radiation treatment for cancer from the prior 5 years. The radiation exposure values that deemed a participant eligible for the study were derived from a review of the literature, consultation with experts, and the motivation to minimize patient risk in voluntary research study participation. If eligibility could not be determined by the first study clinician, a second study clinician reviewed and adjudicated a decision with the first clinician. Cases were also referred for adjudication by a study clinician if participants answered “don’t know” for any of the major procedures listed in the screening tool, or if the research associate noted any participant uncertainty. For adjudication, radiology reports were requested from the participants’ care providers (ie, primary care physicians, cardiologists, oncologists) and electronic health records from clinics or hospitals where participants reported receiving care. The study clinician reviewed these radiology reports within 1 week of receipt, compiled a list of radiation exposure events occurring during the prior year using all available records, and documented specific amounts of radiation per procedure (if available) from the clinical radiology report. The exposure list was returned to the research associate who made necessary adjustments to the screening tool and arrived at an updated total dose of radiation. Reports of undergoing a cardiac stress test often required adjudication by the study physician, as radiation exposure ranges from 2 to 41 mSv depending on the type of cardiac stress test performed.^6,9-11^ If any uncertainty regarding prior exposure remained, a second study clinician reviewed the case, and the 2 clinicians discussed until they achieved consensus.

### Applying the Radiation Safety Screening Tool in the SAGES Substudy

The radiation safety screening tool (Appendix 2) was implemented in a substudy of the ongoing prospective longitudinal SAGES study. During study follow-up, participants were invited to undergo amyloid PET imaging. Participation in the PET substudy was voluntary and involved a separate consent process. Participants who were interested underwent the radiation safety screening process. Once eligibility was determined, participants were scheduled to undergo scanning. All amyloid PET scans were performed at Beth Israel Deaconess Medical Center following standard clinical protocols. In brief, a dose of 10 millicurie (mCi) (within 10%) florbetapir F-18 was injected via intravenous administration. After 1 hour, noncontrast CT images were obtained for attenuation correction and anatomic localization of tracer uptake; CT images were then coregistered and fused with emission PET images.

Because this study involved only a review of the literature and public databases with no individual-level data, as well as completely deidentified secondary data from the SAGES study, this study was deemed not to be human subjects research, and institutional review board approval was not required. The parent SAGES study and amyloid PET substudy obtained written informed consent for study participation according to procedures approved by the institutional review boards of the Beth Israel Deaconess Medical Center and Brigham and Women's Hospital (the 2 study hospitals) and Hebrew SeniorLife (the study coordinating center), all of which are located in Boston, Massachusetts.

## RESULTS

Of the 101 SAGES study participants screened using the radiation safety screening tool (Table 1), individual total exposure doses ranged from 0 to 647 mSv. Participants reported a wide range of radiation exposure levels from previous procedures across numerous categories. The most commonly reported radiologic procedures (mammography and chest, knee, hip, or dental radiographs) were associated with <1 mSv of radiation exposure per procedure (Table 2). Three participants were excluded based on a total exposure of >50 mSv: 1 participant had undergone a prostate artery embolism procedure (>600 mSv), and the other 2 participants were excluded because of cumulative exposure from multiple radiologic procedures (radiographs, CT scans, and/or interventional radiology procedures totaling 12 to 28 procedures per participant). Of the 25 cases sent for study physician adjudication, 56% (n=14) were deemed ineligible for the PET scan because of high exposure, cardiac stress tests, cancer radiation treatment, or a combination of multiple procedures (ranging from 7 to 13 procedures per participant for whom adjudication was required and 4 to 16 procedures for participants for whom adjudication was requested) (Table 3). Among the 84 participants found to be eligible to undergo an amyloid PET scan, 58 participants opted to participate in the SAGES amyloid PET substudy.

**Table 1.** Participant Substudy Eligibility Based on Radiation Safety Screening

	Number of Participants Screened		
Eligibility Status and Substudy^a^ Participation	SAGES I, n=30	SAGES II, n=71	Total, n=101
Eligible for PET scan	21 (70)	63 (89)	84 (83)
Eligible from screening (total dose <30 mSv)	19	54	73
Eligible from screening (total dose <30 mSv), but confirmatory adjudication requested	2	6	8
Adjudication required (total dose 30 to 50 mSv)	0	3	3
Eligible, but declined participation^b^	12 (57)	14 (22)	26 (31)
**Total number of completed PET scans** **^c^**	**9 (30)**	**49 (69)**	**58 (57)**
Noneligible for PET scan	9 (30)	8 (11)	17 (17)
Noneligible from screening (total dose >50 mSv) with confirmatory adjudication	0	3	3
Adjudication required (total dose 30 to 50 mSv)	3	1	4
Adjudication requested (incomplete dose information)	6	4	10

^a^In a substudy of the ongoing SAGES study, participants were invited to undergo amyloid PET imaging.

^b^The denominator used to calculate each percentage in this row is the n of participants eligible for PET scan in each group.

^c^The denominator used to calculate each percentage in this row is the total n of screened participants in each group.

Note: Data are shown as n (%) and as n.

mSv, millisievert; PET, positron emission tomography; SAGES, Successful AGing after Elective Surgery study.

**Table 2.** Radiologic Procedures Reported by 101 Screened Participants

Procedure	Exposure, mSv	Frequency, n (%)
Radiograph		
Dental	0.1	46 (46)
Mammography	0.4	30 (30)
Knee	0.005	25 (25)
Chest	0.12	22 (22)
Hip	0.7	19 (19)
Lumbar spine	1.5	17 (17)
Dual x-ray absorptiometry (DEXA) without computed tomography	0.001	8 (8)
Abdomen	0.7	7 (7)
Shoulder	0.01	7 (7)
Cervical spine	0.2	6 (6)
Thoracic spine	1	6 (6)
Pelvis	0.6	6 (6)
Upper gastrointestinal series	6	5 (5)
Skull	0.1	3 (3)
Computed tomography		
Neck	3	8 (8)
Spine	6	7 (7)
Chest/lung for pulmonary embolism	15	6 (6)
Pelvis	6	6 (6)
Dual x-ray absorptiometry (DEXA) with computed tomography	0.04	2 (2)
Vessel imaging		
Head and/or neck angiography	5	7 (7)
Coronary angiography	16	3 (3)
Calcium scoring	3	1 (1)
Coronary percutaneous transluminal angioplasty	15	1 (1)
Pelvic vein embolization	60	1 (1)
**Nuclear medicine**		
Cardiac stress test^a^	9.4-14.1	10 (10)
Lung nuclear medicine	2	1 (1)
Bone nuclear medicine	6.3	1 (1)
Tumor (18F-FDG) nuclear medicine	14.1	1 (1)

^a^All cardiac stress tests were reviewed for exact radiation exposure.

Note: Some participants reported undergoing some procedures (eg, chest x-ray) more than once in the prior 12 months.

18F-FDG, fluorodeoxyglucose F-18; mSv, millisievert.

**Table 3.** Reasons for Ineligibility Among 101 Participants Screened

Ineligibility Classification	n	Reasons
Noneligible from screening (total dose >50 mSv)	3	• Multiple combined radiographs, CT scans, and interventional procedures (range of 12-28 procedures) • Prostate artery embolism
Adjudication required (total dose 30-50 mSv)	4	• Cardiac stress test • Multiple combined radiographs, CT scans, interventional procedures, and nuclear medicine procedures (range of 7-13 procedures)
Adjudication requested (incomplete dose information)	10	• Cardiac stress test • Multiple combined radiographs, CT scans, and interventional procedures (range of 4-16 procedures)

CT, computed tomography; mSv, millisievert.

## DISCUSSION

We devised a feasible radiation safety screening approach for use in clinical trials and provided a descriptive summary of our workflow and application in our amyloid PET substudy. In our substudy of adults ≥70 years, participants reported a wide range of prior radiation exposure. Seventeen percent of participants were determined to be ineligible because of radiation exposure in the prior 12 months that exceeded the threshold we set for the substudy. The range and magnitude of radiation exposure history detected by the screening tool reinforce the importance of screening research participants for radiation exposure in their personal health history and of considering cumulative doses.

The use of amyloid PET scans in clinical research trials listed on ClinicalTrials.gov has more than doubled during the past decade, growing from 20 studies in 2013 to 54 studies in 2023. With the FDA approval of the anti-amyloid treatments lecanemab (approved January 6, 2023) and donanemab (approved July 2, 2024), the use of amyloid PET scans in research and clinical settings is expected to increase. With the growing use of medical imaging, individual cumulative radiation exposure is expected to increase correspondingly. Thus, ensuring radiation safety and protection will assume greater importance and should remain a priority in the field.^19,33^

Our review found that studies using amyloid PET scans inconsistently used prior radiation exposure screening as an enrollment criterion. The studies that screened for prior radiation exposure followed no strict guidelines and lacked standardization. Such ambiguity in study protocols and the lack of standardization in screening could pose a potential risk for participants.

Ensuring participant safety must be a priority for investigators. First, a careful medical history should be obtained, as certain conditions are associated with a heightened risk of radiation exposure. For instance, individuals with a history of radiation treatment for cancer or a history of cardiac disease,^38,39^ end-stage renal disease,^40,41^ Crohn disease,^42,43^ or endovascular aortic repair^44,45^ tend to have a high cumulative exposure, often >100 mSv.^19^ Second, a review of available medical records by the study physician should be included to determine the most accurate exposure history, particularly if participants are uncertain about their exposure. Our study took a conservative approach in estimating prior radiation exposure and excluded participants who were unable to provide a reliable exposure history. Third, a systematic approach, such as a tracking system incorporated into the electronic health record and local derivation of radiation exposure values at imaging sites, would improve the ability to determine prior radiation exposure, improve participant safety, and help minimize risks associated with increased radiation levels. Last, as imaging becomes more widespread in clinical and research practices, the implications of cumulative radiation dose on study risks will be an increasingly important area for investigation.

A limitation of this study is that our review of current radiation screening practices was based on available data in the ClinicalTrials.gov database, which may not have included all potential studies using amyloid PET. Also, in both ClinicalTrials.gov and the published literature, investigators may have omitted details related to radiation safety screening and exclusion criteria. These omissions, however, underscore the need for uniform reporting requirements. Further, we only reviewed studies using amyloid PET that were similar to the SAGES study (ie, we did not include industry-sponsored drug trials), and we acknowledge that the use of radiation exposure screening may vary across imaging studies, research, and clinical indications.

The lack of an accepted and widely recognized standard for identifying radiation exposure limits for participants in research studies is an important gap in the field. Development of such a standard should be a priority. The standard will need to account for different procedure risks, patient vulnerability factors, and specific study circumstances. Each study will need to adapt or customize the approach to specific circumstances and needs. For example, a more lenient eligibility threshold of prior radiation exposure may be set for research studies in which participants may directly benefit from the procedure. Based on our specific study and cohort, we set a wide range of dosage limits (30 to 50 mSv) that required additional review. In our study, we screened for radiation exposure only in the prior 12 months, as we believed 12 months would be a feasible time interval for participants to accurately recall undergoing a radiologic procedure and for the study team to obtain confirmatory medical records. Nevertheless, cumulative lifetime exposure risks vs short-term exposure risks may be weighed differently. With the growing use of electronic health records, tracking exposure will be more accessible and reliable and can be extended to a longer cumulative exposure period as needed.

Long-term follow-up studies involving radiation exposure from imaging procedures are lacking. While we anticipate that future advances in imaging technology may reduce radiation exposure while also enhancing diagnostic accuracy, trends will need to be carefully monitored to determine if newer protocols or equipment results in an increase or decrease in overall radiation exposure levels.

## CONCLUSION

A comprehensive and standardized method to screen for prior exposure to ionizing radiation in potential participants in clinical research that involves imaging is needed to ensure patient safety. We propose that clinicians and researchers implement a systematic and standardized method adapted for their specific study needs, such as the one we developed for use in our SAGES study. Screening participants for their total dose of radiation exposure prior to enrollment in studies using radiologic procedures will benefit the well-being and safety of research participants. We provided a description of our practical, standardized assessment that includes a comprehensive review of prior radiation exposure. We hope future studies will build on the foundation we have provided.

## Notes

*This article meets the Accreditation Council for Graduate Medical Education and the American Board of Medical Specialties Maintenance of Certification competencies for Patient Care, Medical Knowledge, and Practice-Based Learning and Improvement.*

## Appendix 1. Reviews of the Literature and of ClinicalTrials.Gov

While the benefits of imaging in clinical care outweigh the risks associated with radiation exposure, the risks from elective radiation exposure in research should be carefully considered. We reviewed the literature and the clinical trials listed in ClinicalTrials.gov to determine the current approaches to screening for prior radiation exposure in clinical trials involving amyloid positron emission tomography (PET) scans. This appendix provides the procedures followed for each of these searches, our findings from these reviews, and references.

### PubMed Literature Review

For our PubMed review of the literature, we used the search terms “amyloid PET” and “screening” and filtered for articles written in English and published between January 1, 2013, and August 1, 2024. Our search yielded 3,854 articles. We then applied the filter “clinical trials,” resulting in a total of 127 articles. After initial review, we excluded 6 studies without imaging, 4 studies on systemic amyloidosis, 2 autopsy studies, 2 cardiac studies, and 1 secondary use study. Three additional articles were not available. The study physician reviewed the remaining 109 articles for the inclusion of any radiation safety guidelines or screening protocols based on prior radiation exposure.

Only 2 of the 109 studies that involved amyloid PET imaging mentioned specific radiation safety guidelines. Rowe et al stated “total radiation exposure from this study fell within the Australian guidelines for research radiation exposure for subjects older than 60 years,”^1^ and Florian et al noted the following exclusion criterion: “contraindication to or inability to tolerate a PET scan (includes current or recent participation in any procedures involving radioactive agents such that the total radiation dose exposure to the patient in any given year would exceed the local country limits of annual and total dose).”^2^ Seven studies were less specific, excluding patients who had been administered a radiopharmaceutical within 10 radioactive half-lives before study tracer administration,^3-5^ a history of previous PET scans,^6^ contraindication for magnetic resonance imaging or PET scan,^7,8^ or history of cancer within 5 years.^9^ Consequently, 92% of the reviewed studies (n=100) did not include any specific details of radiation exposure as a criterion for study participation.

### ClinicalTrials.gov Study Review

We reviewed the ClinicalTrials.gov database to identify planned or active clinical trials using PET scans. Using the search term “amyloid PET,” we identified 567 studies that had been posted to the site as of October 3, 2024. To select for studies relevant to our target population, we excluded studies that had a start date before 2018, were affiliated with industry, were not conducted in the United States, or did not include older adults (n=462). We excluded an additional 13 trials because the target conditions did not include brain diseases (eg, cardiac amyloidosis, breast cancer). A trained research assistant reviewed the remaining 92 trials and classified them into 4 categories based on the following radiation safety exclusions: (1) specific radiation exposure limit based on prior procedures, (2) nonspecific or unspecified prior radiation exposure limit, (3) exclusion for specific radiologic treatments or procedures, or (4) pregnancy-related exclusion.

Among the 92 studies listed on ClinicalTrials.gov, 35 (38%) did not include any exclusion criteria relating to previous radiation exposure in the study description (Appendix Table 1). Of the studies that included exclusion criteria, only 1 study (1%) specified a radiation exposure threshold for exclusion. Six studies (7%) excluded participants based on a history of undergoing a specific radiologic treatment or procedure, such as active treatment for cancer, chemotherapy, or radiation treatment within the prior year. A low degree of radiation exposure was allowed for pregnant participants in 18 (20%) trials, but pregnancy alone was specified as an exclusion criterion for 24 (26%) trials. Eight studies mentioned an exposure limit but did not provide specific values (9%). Appendix Table 2 provides a list of all the studies, grouped by exclusion criteria.

**Appendix Table 1.** Summary of Radiation Exposure Exclusion Criteria for ClinicalTrials.gov-Registered Alzheimer Disease Studies Using Amyloid Positron Emission Tomography, n=92

Exclusion Criterion	Number of Studies
Specific mSv or rem values for exposure	1 (1)
Specific radiologic treatment/procedure	6 (7)
Pregnancy with a low-degree exposure limit (mSv or rem and vague terms)	18 (20)
Pregnancy only	24 (26)
Exposure limit without specific mSv or rem values	8 (9)
None	35 (38)

Note: Data are presented as n (%).

mSv, millisievert; rem, roentgen equivalent man.

**Appendix Table 2.** ClinicalTrials.gov-Registered Alzheimer Disease Studies Using Amyloid Positron Emission Tomography Grouped by Radiation Exposure Exclusion Criteria, n=92

Exclusion Criterion	NCT Number	Clinical Trial Name
Specific mSv or rem values for exposure, n=1	NCT04804241	Senicapoc in Alzheimer's Disease
Specific radiologic treatment/procedure, n=6	NCT03053908	Orexin and Tau Pathology in Cognitively Normal Elderly
	NCT03644043	Evidence Amyloid Scan EEG Study
	NCT03466736	Enriching Clinical Trials Requiring Amyloid Positivity With Practice Effects
	NCT04080544	Cognitive Decline and Alzheimer's Disease in the Dallas Lifespan Brain Study
	NCT03926702	Tau Imaging With JNJ067
	NCT02359864	Study of Low Dose Whole Brain Irradiation in the Treatment of Alzheimer's Disease
Pregnancy with a low-degree exposure limit (mSv or rem and vague terms), n=18	NCT03319810	Effect of IVIG on Cerebral and Retinal Amyloid in Mild Cognitive Impairment Due to Alzheimer Disease
	NCT04057807	Peripheral Benzodiazepine Receptors (PBR28) Brain PET Imaging With Lipopolysaccharide Challenge for the Study of Microglia Function in Alzheimer's Disease
	NCT03412604	Effect of Modulating Gamma Oscillations Using tACS
	NCT03699644	Multimodal Ocular Imaging in Neurodegeneration
	NCT03706261	Alzheimer's PET Imaging in Racially/Ethnically Diverse Adults
	NCT03958630	PET Imaging of Neuroinflammation in Neurodegenerative Diseases Via a Novel TSPO Radioligand
	NCT03880240	Gamma Induction for Alzheimer's Disease
	NCT02414178	F 18 T807 Tau PET Imaging in Dominantly Inherited Alzheimer's Network (DIAN Project)
	NCT05164536	Plasma P-tau2017 and Quantitative Amyloid PET Imaging
	NCT04579120	Neuroimaging in Healthy Aging and Senile Dementia (HASD_IND)
	NCT04629547	Sleep Trial to Prevent Alzheimer's Disease
	NCT05331144	Impact of Intensive Treatment of SBP on Brain Perfusion, Amyloid, and Tau (IPAT Study)
	NCT05582200	Selective PET Imaging of Astrocytes and Microglia in Alzheimer's Disease
	NCT05464368	R21 Roche: 3-Way Tau Tracers in AD
	NCT03860857	MRI and PET Biomarkers for Cognitive Decline in Older Adults
	NCT04720001	Florbetaben PET Imaging in PPA
	NCT04840979	Discovery and Validation of Genetic Variants Affecting Microglial Activation in Alzheimer's Disease
	NCT05731440	Fluselenamyl – Beta Amyloid PET Imaging for Alzheimer Disease
Pregnancy only, n=24	NCT03019757	Distinguishing Between Alzheimer's Disease, Lewy Body Dementia, and Parkinson's Disease
	NCT04871074	The Synapse Project
	NCT03919669	A Longitudinal Evaluation of a Radiotracer for Use in Tau Tracking
	NCT04274998	Neuroinflammation Imaging in AD
	NCT04468659	AHEAD 3-45 Study: A Study to Evaluate Efficacy and Safety of Treatment With Lecanemab in Participants With Preclinical Alzheimer's Disease and Elevated Amyloid and Also in Participants With Early Preclinical Alzheimer's Disease and Intermediate Amyloid
	NCT03981380	11C-PIB PET Study in MESA at Columbia University
	NCT03333551	Cardiac Uptake of 18F Florbetapir in Patients Undergoing Chemotherapy
	NCT04497168	Citalopram as a Posterior Cortical Protective Therapy in Parkinson Disease
	NCT04994847	APOE in the Predisposition to, Protection From, and Prevention of Alzheimer's Disease
	NCT04847453	Venetoclax, MLN9708 (Ixazomib Citrate) and Dexamethasone for the Treatment of Relapsed or Refractory Light Chain Amyloidosis
	NCT05361382	Head-to-Head Harmonization of Tau Tracers in Alzheimer's Disease
	NCT05269394	Dominantly Inherited Alzheimer Network Trial: An Opportunity to Prevent Dementia. A Study of Potential Disease Modifying Treatments in Individuals With a Type of Early Onset Alzheimer's Disease Caused by a Genetic Mutation (DIAN-TU)
	NCT03816228	Imaging of Brain Structural/Functional Connectivity and Amyloid and Tau Lesions in APOE4 Carriers.
	NCT05552157	A Study of Potential Disease Modifying Treatments in Individuals at Risk for or With a Type of Early Onset AD Caused by a Genetic Mutation
	NCT03757910	Brain Imaging in the Diabetes Prevention Program Outcomes Study
	NCT04055532	Biomarkers in Neurodegenerative Diseases
	NCT04680130	Clinico-Pathologic-Genetic-Imaging Study of Neurodegenerative and Related Disorders
	NCT04426539	New IDEAS: Imaging Dementia-Evidence for Amyloid Scanning Study
	NCT04098666	Metformin in Alzheimer's Dementia Prevention
	NCT04002674	Impact of Nilotinib on Safety, Tolerability, Pharmacokinetics and Biomarkers in Dementia With Lewy Bodies
	NCT04118764	Non-invasive Blood-brain Barrier Opening in Alzheimer's Disease Patients Using Focused Ultrasound
	NCT04629495	Rapamycin – Effects on Alzheimer's and Cognitive Health
	NCT05617014	Alzheimer's Disease Neuroimaging Initiative 4
	NCT05776641	Gamma Light and Sound Stimulation to Prevent Dementia in Cognitively Normal People at Risk for Alzheimer's Disease
Exposure limit without specific mSv or rem values, n=8	NCT03282916	Anti-viral Therapy in Alzheimer's Disease
	NCT03663387	PET Measures of CSF Clearance in Preclinical Alzheimer's Disease
	NCT03744312	Imaging Inflammation in Alzheimer's Disease With 11C-ER176
	NCT03913637	Facilitating Optimal Routines in Aging
	NCT04510168	Accelerated Non-Atherosclerotic Brain Arterial Aging Relationship to Alzheimer's Disease
	NCT04576793	Longitudinal Imaging of Microglial Activation in Different Clinical Variants of Alzheimer's Disease
	NCT04710030	Valacyclovir for Mild Cognitive Impairment
	NCT04134923	Imaging Biomarkers in Preclinical and Symptomatic AD
None, n=35	NCT03503331	UAB Alzheimer's Disease Center Core Cohort – Imaging Substudy
	NCT03278119	Sleep Aging and Risk for Alzheimer's 2.0
	NCT03582488	Longitudinal Imaging Biomarkers of Disease Progression in DLB
	NCT04041895	Detecting Probable Alzheimer's Disease From Speech Using Linguistical Analysis
	NCT03814603	The Sleep Amyloid, Slow WAve Race and Ethnicity Study
	NCT03641768	Risk Prediction for Alzheimer Dementia With Brain Imaging and Genetics
	NCT03899844	Study to Evaluate Amyloid in Blood and Imaging Related to Dementia
	NCT03999879	PhosphoRus, Proton Imaging and Amyloid BuRdEn (PREPARE) ON AMYLOID BURDEN AND COGNITION
	NCT03718494	Continuation of The Kronos Early Estrogen Prevention Study (KEEPS)
	NCT03821857	Sex-Specific Effects of Endocrine Disruption on Aging and Alzheimer's Disease
	NCT03954210	SIESTA: Sleep Intervention to Enhance Cognitive Status and Reduce Beta Amyloid
	NCT04140708	Effects of Exercise on Glymphatic Functioning and Neurobehavioral Correlates in Parkinson's Disease
	NCT03560960	Multi-Center Development of a Novel Diagnostic Test for Alzheimer's Disease
	NCT04251130	Linking Tau PET to Medial Temporal Lobe Subregions With High Resolution MRI
	NCT04263337	NFL LONG Prospective Study
	NCT03809351	UAB Alzheimer's Disease Center Core Cohort - Tau Imaging Substudy
	NCT04032626	MCLENA-1: A Clinical Trial for the Assessment of Lenalidomide in Amnestic MCI Patients
	NCT04403165	Locus-coeruleus Function in Normal Elderly and AD Risk
	NCT05785871	Hypertension, Brain Clearance, and Markers of Neurodegeneration
	NCT04818255	STIM+: PET Biomarker Education & Disclosure
	NCT04786223	Targeting Neuroinflammation as a Contributing Pathology in Alzheimer's Disease Dementia
	NCT04309500	SHARE(D) Stage II: Alzheimer's Risk Disclosure Protocol Piloting
	NCT05446805	Depression and Driving
	NCT05094271	Is Obstructive Sleep Apnea Important in the Development of Alzheimer's Disease?
	NCT05138848	Time-in-bed Restriction in Older Adults With Sleep Difficulties With and Without Risk for Alzheimer's Disease
	NCT05040217	A Clinical Trial of AAV2-BDNF Gene Therapy in Early Alzheimer's Disease and Mild Cognitive Impairment
	NCT05469009	Safety and Feasibility of Exablate Blood-Brain Barrier Disruption for Mild Cognitive Impairment or Mild Alzheimer's Disease Undergoing Aduhelm Therapy.
	NCT05758493	Characterizing Iodine-124 Evuzumitide (AT-01) in Systemic Amyloidosis
	NCT05584241	Behavioral Change Following Alzheimer's Disease (AD) Biomarker Disclosure
	NCT05606341	Innate Immunity Stimulation Via TLR9 in Early AD
	NCT05820932	Predicting Cognitive Decline From Androgen Deprivation Therapy
	NCT05457998	BioFINDER-Brown: Examination of Alzheimer's Disease Biomarkers
	NCT04899180	Prevalence of Transthyretin Cardiac Amyloidosis in Clinically Significant Aortic Stenosis
	NCT05882344	Deep Brain Stimulation for Alzheimer's
	NCT04082611	The PREVENTION Trial: Precision Recommendations to Optimize Neurocognition

mSv, millisievert; NCT, national clinical trial; rem, roentgen equivalent man.
